# The Clinicopathological Significance and Prognostic Value of Androgen Receptor in Endometrial Carcinoma: A Meta-Analysis

**DOI:** 10.3389/fonc.2022.905809

**Published:** 2022-06-22

**Authors:** Xirong Wu, Xiuli Zhong, Xiaoqing Huo, Junrong Zhang, Xiaoqing Yang, Yuquan Zhang

**Affiliations:** Department of Gynecology and Obstetrics, Affiliated Hospital of Nantong University, Nantong, China

**Keywords:** androgen receptor, clinicopathological, prognosis, endometrial cancer, meta-analysis

## Abstract

**Background:**

The role of androgen receptor (AR) in evaluating the prognosis of patients with endometrial cancer (EC) remains controversial. Here, we performed a meta-analysis to assess whether AR expression improves EC survival outcomes.

**Methods:**

We searched related articles published before August 2021 in PubMed, EMBASE, and Web of Science. The association between AR expression and patient prognosis was estimated with hazard ratios (HRs) and odds ratios (ORs) with their corresponding 95% confidence intervals (95% CIs). The review is registered on PROSPERO, registration number: CRD42021268591.

**Results:**

Ten studies including 1,485 patients were enrolled in the meta-analysis. The results showed that AR expression in EC tissues was associated with a better survival in crude analyses (HR = 1.63, 95% CI = 1.32–2.02, P < 0.001). However, no significant relation was found after the adjustment of the confounding factors (HR = 1.68, 95% CI = 0.75–3.75, P = 0.205). In subgroup analyses, grade 1–2 disease, stage I–II disease, negative lymph node status, and lack of the lymphovascular invasion were more common in AR-positive groups (OR = 0.47, 0.48, 0.37, and 0.57; 95% CI = 0.45–0.62, 0.35–0.65, 0.24–0.56, and 0.37–0.89). Furthermore, AR expression was more common in endometrioid cancers (OR = 2.39, 95% CI = 1.79–3.20).

**Conclusions:**

AR expression is significantly associated favorable characteristics including low-grade disease, early-stage disease, negative lymph node status, and lack of the lymphovascular invasion and a specific histology—endometrioid cancer. However, AR is not an independent prognostic factor.

## Introduction

Endometrial cancer (EC) is the most common gynecologic malignancy and continues to increase by about 1% per year (1). During 2021, almost 66,570 new cases of uterine corpus cancer and 12,940 deaths are projected to occur due to this cancer in the United States (2).

An excess-estrogen environment is linked with EC development, especially type I cancer (3). As the main source of estrogen especially in postmenopausal women, the importance of androgens in EC has been recognized for the last decades. In addition, androgen receptor (AR) also has been evaluated for its prognostic power in EC. In some studies, AR expression has been reported to be associated with better survival in patients with EC (4–8), whereas the better prognosis was not noted in other studies (9, 10). For explaining better prognosis in patients with EC, some investigators thought that the heterogeneity of histology resulted in the different patient survival of EC. However, the identical findings were not identified (5, 8–10).

With the aim of disentangling these controversial issues, we present a systematic review and meta-analysis to evaluate the association between the AR expression and the prognosis of patients with EC.

## Materials and Methods

This research was conducted according to Preferred Reporting for Systematic Reviews and Meta-Analyses (PRISMA) principles.

### Literature Search

We performed a comprehensive search in PubMed, EMBASE, and Web of Science. The search terms included “endometrial cancer” or “endometrial carcinoma” or “endometrial neoplasms” in combination with “androgen receptors”. Titles and abstracts were checked to identify potential eligible articles by two researchers, who then reviewed full texts. In addition, the references of included articles were checked manually for more related studies.

### Inclusion and Exclusion Criteria

The inclusion criteria were as follows: (1) studies published in English; (2) studies on EC that confirmed by histopathological examination; (3) studies assessing AR expression with positive or negative labels; and (4) studies comparing the relationship between AR and clinic-pathological characteristics or prognosis. However, we excluded studies as follows: (1) studies based on animals or *in vitro* experiments; (2) review articles, meta-analyses, letters, or case reports; and (3) non-English literature.

### Data Extraction

For included articles, two investigators independently extracted the related data using a fixed form. The form included the name of the first author, the year of publication, age, the expression level of AR, clinic-pathological characteristics, hazard ratios (HRs), and 95% confidence intervals (CIs) for survival analysis. If the HRs and 95% CIs could not be acquired directly, then they were estimated from Kaplan–Meier curves using the method described by Parmar et al. (11). Two studies (6, 7) were excluded because of the significant difference between the estimated and actual HR. Any disagreements were resolved by discussion and consultation with the third author.

### Quality Assessment

The guidelines from the Newcastle-Ottawa Scale (NOS) criteria were used to evaluated the quality of studies (12). The NOS criteria included three domains: (1) selection: 0–4; (2) comparability: 0–2; and (3) exposure or outcomes: 0–3. Good quality was considered when the NOS scores ≥6.

### Statistical Analysis

Dichotomous data eligible in each research were shown as a odds ratio (OR) with its 95% CI.

Moreover, the pooled HRs and 95% CIs were calculated to evaluate the associations between AR and prognosis of patients with EC. Heterogeneity between studies was assessed using *I^2^* (13). If *I^2^* >50%, substantial heterogeneity was considered and the random effects model was implemented. When *I^2^* ≤50%, the fixed effect model was used in this meta-analysis.

Publication and selection bias was investigated by funnel plots and the Egger and Begg test. All analyses were performed in STATA software, and P < 0.05 was considered statistically significant.

## Results

### Study Search

**Figure 1:**
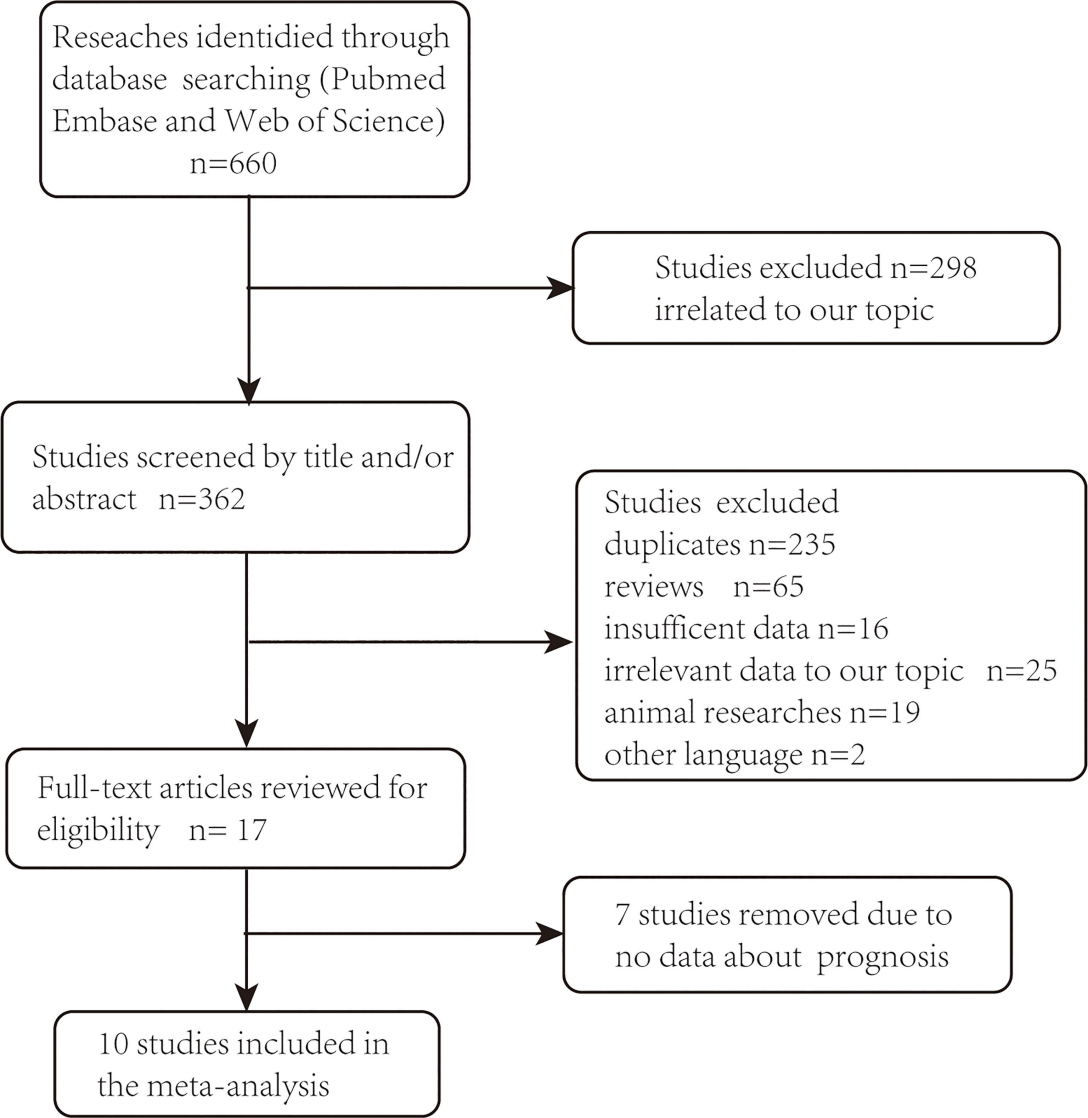
PRISMA flowchart of eligible studies selection process.

A total of 660 studies were identified. After removal of 298 duplicates, 362 records were checked based on title and/or abstract and 17 studies remained. The full texts of remaining articles were further assessed for more details, and seven articles were excluded for the lack of data on prognosis or clinicopathological characteristics. Finally, 10 studies including 1,485 patients were enrolled in the meta-analysis (**Figure 1**). The main characteristics of included studies are shown in **Table 1**. Briefly, all of the articles investigated the association between AR and various clinicopathologic factors (4–10, 14–16), among which five of them further performed survival analysis (4–8).

**Table 1:** Characteristics of the included studies.

Study	Year	Country	No. of Cases	Examination Methods	Clinic-Pathological Characteristics			
					AR Positive(%)	Histological Type	Disease-Progressive Indicators	Survival Analyses
Abu Shahin et al.	2021	Jordan	52	IHC	28/52 (53.8%)	Endometrioid Serous Clear-cell	FIGO stage Grade Lymph node status	NA
Nisar et al.	2020	Pakistan	54	IHC	29/54 (53.7%)	Endometrioid Serous Clear-cell Carcinosarcoma	Grade Lymphovascular invasion Myometrial invasion	NA
Hashmi et al.	2018	Pakistan	103	IHC	18/89	Endometrioid Serous Clear-cell Carcinosarcoma	FIGO stage Grade Lymphovascular invasion Myometrial invasion Cervical invasion Lymph node status	NA
Park et al.	2018	Korea	51	IHC	30/51 (58.8%)	NA	Grade Myometrial invasion	DFS+OS
Roy et al.	2017	India	25	IHC	14/25 (56.0%)	Stromal sarcoma	Grade	NA
Mahdi et al.	2017	USA	261	IHC	135/261 (51.7%)	Endometrioid Mucinous Serous Clear-cell Carcinosarcoma	FIGO stage Grade Lymphovascular invasion Lymph node status	OS
Zadeh et al.	2017	USA	50	IHC	27/50 (54%)	Endometrioid Serous Clear-cell Carcinosarcoma	Grade	NA
Kamal et al.	2016	UK	85	IHC	54/86 (62.8%)	Endometrioid Serous Clear-cell Carcinosarcoma	FIGO stage Grade Lymphovascular invasion Myometrial invasion Cervical invasion	DFS
Tangen et al.	2016	Norway	718	IHC	447/718 (62.3%)	Endometrioid Serous Clear-cell Carcinosarcoma Adeosquamous Undifferentiated/other	FIGO stage Grade Lymph node status	DSS
Tanaka et al.	2015	Japan	86	IHC	65/86 (75.6%)	NA	FIGO stage Grade Lymphovascular invasion Myometrial invasion Lymph node status	PFS

IHC, immunohistochemistry; DFS, disease-free survival; DSS, disease-specific survival; OS, overall survival; PFS, progression-free survival; NA, not applicable.

### Impact of AR on EC Prognosis

**Figure 2:**
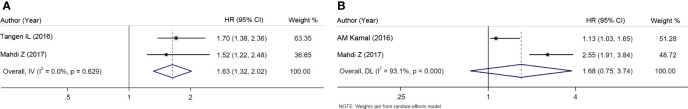
Meta-analysis of the association between AR and patient survival. **(A)** Univariate survival analysis. **(B)** Multivariate survival analysis.

Given the effect of the confounding factors, a stratified analysis was conducted on the subsets of survival analysis. The two available studies on univariate survival analysis suggest that AR overexpression predicted a favorable survival (HR = 1.63, 95% CI = 1.32–2.02, P < 0.001; **Figure 2A**) (5, 8). However, in two studies using multivariate survival analysis (4, 8), no significant relation was observed after adjustment for potential confounding factors (HR = 1.68, 95% CI = 0.75–3.74, P = 0.205; **Figure 2B**).

### Clinicopathologic Characteristics of AR Expression in EC

**Figure 3:**
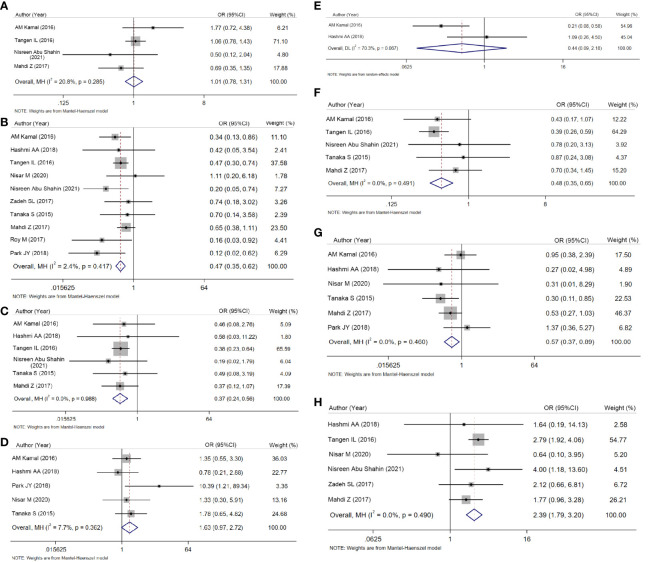
Forest plots for ORs and 95% CIs to compare clinicopathologic characteristics. **(A)** Age. **(B)** Grade. **(C)** Lymph node status. **(D)** Myometrial invasion. **(E)** Cervical invasion. **(F)** Stage (I + II vs. III + IV). **(G)** Lymphovascular invasion. **(H)** Histological type (I vs. II).

Finally, we evaluated clinicopathologic characteristics between AR-positive and AR-negative groups. In crude analyses, low grade (OR = 0.466, 95% CI = 0.352–0.618, P < 0.001; **Figure 3B**), negative lymph nodes (OR = 0.367, 95% CI = 0.239–0.564, P < 0.001; **Figure 3C**), FIGO stage I–II disease (OR = 0.480, 95% CI = 0.353–0653, P < 0.001; **Figure 3F**), and negative lymphovascular invasion (OR = 0.572, 95% CI = 0.368–0.890, P = 0.013; **Figure 3G**) were more common in AR-positive group. However, the associations between AR expression and age, myometrial invasion and cervical invasion were not statistically significant (**Figures 3A, D, E**; P=0.941, P=0.063, and P=0.317, respectively).

In terms of histology, crude analysis showed type I cancers were more frequent in AR-positive group (OR = 2.393, 95% CI = 1.789–3.202, P < 0.001; **Figure 3H**).

### Publication Bias Assessment

Begg’s funnel plot was conducted to assess the publication bias of included studies and no evidence of publication bias was seen (**Supplementary Figure 1**).

## Discussion

The role of AR in EC has been widely discussed for decades. However, the prognostic usefulness of AR is still controversial. This is the first systematic review with meta-analysis to examine the effect of AR on survival outcomes in patients with EC. We found that AR expression imparts a better survival outcome. The effect on better prognosis was consistently observed in subgroup analyses according to clinicopathologic characteristics. EC is a biologically and histologically diverse group of neoplasms characterized by a dualistic model of pathogenesis. Unlike type II EC, type I endometrial tumors usually portend a less aggressive clinical course (17). Our meta-analytic results showed that AR may have favorable characteristics of type I EC including early-stage disease, low-grade disease, negative lymph node status, and lack of the lymphovascular invasion. Indeed, we found that the expression of AR significantly increased in type I cancers. These findings mean that AR plays a crucial role in type I rather than type II cancers.

Notably, numerous studies have also examined the potential role of androgens as risk factors for EC. In addition, most of them claimed to have found that elevated serum testosterone level increased EC risk (18–21). It is tempting to speculate that AR is one of negative prognostic factors in EC. However, our meta-analysis reports that AR expression is a favorable prognostic indicator. It is well known that testosterone can be metabolized by aromatase and 5α-reductase to estradiol and dihydrotestosterone (DHT), respectively (22). An excess-estrogen environment can trigger the development and progression of EC, especially for type I. It is reported that the inhibition of aromatase activity has been applied to the treatment of EC. A retrospective cohort study recently reported longer PFS (HR = 0.23; 95% CI = 0.04–1.27) and OS (HR = 0.11; 95% CI = 0.01–1.36) in patients receiving aromatase inhibitors (AIs) (23). On the other hand, Hashimoto et al. have reported that DHT could inhibit the proliferation of EC cells (24). Consistent with these findings, the results in our study indirectly show that the conversion of testosterone to DHT and further activation of AR by DHT inhibit the continuum of EC progression.

Two of the included articles performed multivariate Cox survival analysis including tumor stage, myometrial invasion, race, BMI, diabetes, and AR, ER, and PR expression (4, 8). This meta-analysis integrated these disparate results, and the data in these studies were not always consistent. This might be ascribable to the following factors. First, AR signaling may have both oncogenic and tumor suppressive roles. In mouse models of type I EC, short-term enzalutamide treatment, an inhibitor of AR signaling, reduced endometrial tumor burden and increased cancer cell apoptosis in a dose-dependent way. However, enzalutamide increased the incidence of invasive and metastatic tumor (25). Oncogenic role of AR may be more involved in EC initiation. Later stages of invasion and metastasis in EC maybe partly due to inactivation of cancer suppressive AR signaling. Second, the histological structures and the carcinogenesis are different in type I and II cancers. Type I cancers are hormone-dependent. Our meta-analytic results showed AR expression was more likely to be observed in type I cancers. This might indicate that the impact of AR may be more inclined to type I EC. Further studies should also focus on the evaluation of the role of AR in type I cancers. Third, studies in the analysis employed different antibodies and cutoff values that led to variations of the results. Fourth, the numbers of patients and outcome events were small that implied poor statistical precision.

This is the first meta-analysis to uncover the prognostic value of AR in patients in EC. However, some limitations in our study should be mentioned. First, some of the studies in the meta-analyses did not mention any preoperative and/or postoperative therapies. Radiotherapy and/or chemotherapy are usually offered for those in advanced stage (26, 27). Such variations in treatment modalities must have an impact on the prognosis and prognostic analyses. Second, the numbers of patients and outcome events were mostly small implying poor statistical precision. Third, heterogeneity was evident among the included studies with respect to the specifics of staining methods, cutoff values, and so on.

In summary, the results from this meta-analysis suggested that AR may be useful prognostic biomarkers for EC. Further well-designed, multi-center, and larger-scale trials are needed to confirm our findings.

## Author Contributions

XW conceived and designed the study, interpreted the data, and drafted the manuscript. XY and YZ designed and revised the manuscript. XZ and JZ selected the articles. XZ and XH retrieved the data. All authors contributed to the article and approved the submitted version.

## Conflict of Interest

The authors declare that the research was conducted in the absence of any commercial or financial relationships that could be construed as a potential conflict of interest.

## Publisher’s Note

All claims expressed in this article are solely those of the authors and do not necessarily represent those of their affiliated organizations, or those of the publisher, the editors and the reviewers. Any product that may be evaluated in this article, or claim that may be made by its manufacturer, is not guaranteed or endorsed by the publisher.

## References

[1] Lortet-Tieulent J Ferlay J Bray F Jemal A. International Patterns and Trends in Endometrial Cancer Incidence, 1978-2013. J Natl Cancer Institute (2018) 110(4):354–61. doi: 10.1093/jnci/djx214 29045681

[2] Siegel RL Miller KD Fuchs HE Jemal A. Cancer Statistics, 2021. CA: Cancer J Clin (2021) 71(1):7–33. doi: 10.3322/caac.21654 33433946

[3] Kaaks R Lukanova A Kurzer MS. Obesity, Endogenous Hormones, and Endometrial Cancer Risk: A Synthetic Review. Cancer epidemiology Biomarkers Prev Publ Am Assoc Cancer Research cosponsored by Am Soc Prev Oncol (2002) 11(12):1531–43. 12496040

[4] Kamal AM Bulmer JN DeCruze SB Stringfellow HF Martin-Hirsch P Hapangama DK. Androgen Receptors Are Acquired by Healthy Postmenopausal Endometrial Epithelium and Their Subsequent Loss in Endometrial Cancer Is Associated With Poor Survival. Br J cancer (2016) 114(6):688–96. doi: 10.1038/bjc.2016.16 PMC4800292 26930451

[5] Tangen IL Onyango TB Kopperud R Berg A Halle MK Øyan AM. Androgen Receptor as Potential Therapeutic Target in Metastatic Endometrial Cancer. Oncotarget (2016) 7(31):49289–98. doi: 10.18632/oncotarget.10334 PMC5226508 27384477

[6] Park JY Baek MH Park Y Kim YT Nam JH. Investigation of Hormone Receptor Expression and Its Prognostic Value in Endometrial Stromal Sarcoma. Virchows Archiv an Int J pathol (2018) 473(1):61–9. doi: 10.1007/s00428-018-2358-5 29869299

[7] Tanaka S Miki Y Hashimoto C Takagi K Doe Z Li B. The Role of 5α-Reductase Type 1 Associated With Intratumoral Dihydrotestosterone Concentrations in Human Endometrial Carcinoma. Mol Cell endocrinol (2015) 401:56–64. doi: 10.1016/j.mce.2014.11.022 25475427

[8] Mahdi Z Abdulfatah E Pardeshi V Hassan O Schultz D Morris R. The Impact of Androgen Receptor Expression on Endometrial Carcinoma Recurrence and Survival. Int J gynecol Pathol Off J Int Soc Gynecol Pathologists (2017) 36(5):405–11. doi: 10.1097/PGP.0000000000000355 28277313

[9] Hashmi AA Hussain ZF Qadri A Irfan M Ramzan S Faridi N. Androgen Receptor Expression in Endometrial Carcinoma and Its Correlation With Clinicopathologic Features. BMC Res notes (2018) 11(1):289. doi: 10.1186/s13104-018-3403-9 29747687 PMC5946473

[10] Nisar M Mushtaq S Hassan U Akhtar N Azma M. Androgen Receptor Expression In Endometrial Carcinoma And Its Correlation With Estrogen Receptor And Progesterone Receptor And Clinicopathological Findings. J Ayub Med College Abbottabad JAMC (2020) 32(2):160–4. 32583986

[11] Parmar MK Torri V Stewart L. Extracting Summary Statistics to Perform Meta-Analyses of the Published Literature for Survival Endpoints. Stat Med (1998) 17(24):2815–34. doi: 10.1002/(SICI)1097-0258(19981230)17:24<2815::AID-SIM110>3.0.CO;2-8 9921604

[12] Stang A. Critical Evaluation of the Newcastle-Ottawa Scale for the Assessment of the Quality of Nonrandomized Studies in Meta-Analyses. Eur J Epidemiol (2010) 25(9):603–5. doi: 10.1007/s10654-010-9491-z 20652370

[13] Higgins JP Thompson SG Deeks JJ Altman DG. Measuring Inconsistency in Meta-Analyses. BMJ (Clinical Res ed) (2003) 327(7414):557–60. doi: 10.1136/bmj.327.7414.557 PMC192859 12958120

[14] Abu Shahin N Aladily T Abu Alhaj N Al-Khader A Alqaqa S Aljaberi R. Differential Expression of Androgen Receptor in Type I and Type II Endometrial Carcinomas: A Clinicopathological Analysis and Correlation With Outcome. Oman Med J (2021) 36(2):e245. doi: 10.5001/omj.2021.53 33833869 PMC8015675

[15] Zadeh SL Duska LR Mills AM. Androgen Receptor Expression in Endometrial Carcinoma. Int J gynecol Pathol Off J Int Soc Gynecol Pathologists (2018) 37(2):167–73. doi: 10.1097/PGP.0000000000000401 28582344

[16] Roy M Kumar S Bhatla N Ray MD Kumar L Jain D. Androgen Receptor Expression in Endometrial Stromal Sarcoma: Correlation With Clinicopathologic Features. Int J gynecol Pathol Off J Int Soc Gynecol Pathologists (2017) 36(5):420–7. doi: 10.1097/PGP.0000000000000353 28114189

[17] Buchanan EM Weinstein LC Hillson C. Endometrial Cancer. Am Family physician (2009) 80(10):1075–80. 19904892

[18] Teng F Ma X Yu X Yan Y Zhao J Gao J. High Serum Androgen and Insulin Concentrations Increase the Tendency of Endometrial Carcinoma. J Cancer (2020) 11(19):5656–64. doi: 10.7150/jca.46391 PMC7477453 32913460

[19] Michels KA Brinton LA Wentzensen N Pan K Chen C Anderson GL. Postmenopausal Androgen Metabolism and Endometrial Cancer Risk in the Women's Health Initiative Observational Study. . JNCI Cancer Spectr (2019) 3(3):pkz029. doi: 10.1093/jncics/pkz029 31321379 PMC6620792

[20] Audet-Walsh E Lépine J Grégoire J Plante M Caron P Têtu B. Profiling of Endogenous Estrogens, Their Precursors, and Metabolites in Endometrial Cancer Patients: Association With Risk and Relationship to Clinical Characteristics. J Clin Endocrinol Metab (2011) 96(2):E330–9. doi: 10.1210/jc.2010-2050 21147881

[21] Lukanova A Lundin E Micheli A Arslan A Ferrari P Rinaldi S. Circulating Levels of Sex Steroid Hormones and Risk of Endometrial Cancer in Postmenopausal Women. Int J cancer (2004) 108(3):425–32. doi: 10.1002/ijc.11529 14648710

[22] McNamara KM Moore NL Hickey TE Sasano H Tilley WD. Complexities of Androgen Receptor Signalling in Breast Cancer. Endocrine-related Cancer (2014) 21(4):T161–81. doi: 10.1530/ERC-14-0243 24951107

[23] Paleari L Rutigliani M Siri G Provinciali N Colombo N Decensi A. Aromatase Inhibitors as Adjuvant Treatment for ER/PgR Positive Stage I Endometrial Carcinoma: A Retrospective Cohort Study. Int J Mol Sci (2020) 21(6):2227–33. doi: 10.3390/ijms21062227 PMC7139521 32210157

[24] Hashimoto C Miki Y Tanaka S Takagi K Fue M Doe Z. 17β-Hydroxysteroid Dehydrogenase Type 2 Expression Is Induced by Androgen Signaling in Endometrial Cancer. Int J Mol Sci (2018) 19(4):1139–52. doi: 10.3390/ijms19041139 PMC5979403 29642629

[25] Koivisto CS Parrish M Bonala SB Ngoi S Torres A Gallagher J. Evaluating the Efficacy of Enzalutamide and the Development of Resistance in a Preclinical Mouse Model of Type-I Endometrial Carcinoma. Neoplasia (New York NY) (2020) 22(10):484–96. doi: 10.1016/j.neo.2020.07.003 PMC7452078 32818842

[26] Hoskins PJ Swenerton KD Pike JA Wong F Lim P Acquino-Parsons C. Paclitaxel and Carboplatin, Alone or With Irradiation, in Advanced or Recurrent Endometrial Cancer: A Phase II Study. J Clin Oncol Off J Am Soc Clin Oncol (2001) 19(20):4048–53. doi: 10.1200/JCO.2001.19.20.4048 11600606

[27] Aalders J Abeler V Kolstad P Onsrud M. Postoperative External Irradiation and Prognostic Parameters in Stage I Endometrial Carcinoma: Clinical and Histopathologic Study of 540 Patients. Obstetrics gynecology (1980) 56(4):419–27. 6999399

