# Supplementary material for: The Clinicopathological Significance and Prognostic Value of Androgen Receptor in Endometrial Carcinoma: A Meta-Analysis
Source: Front Oncol. 2022 Jun 22;12:905809. doi: 10.3389/fonc.2022.905809 (PMC9257049; doi:10.3389/fonc.2022.905809)
Supplement: Supplementary file 1 [file DataSheet_1.docx]

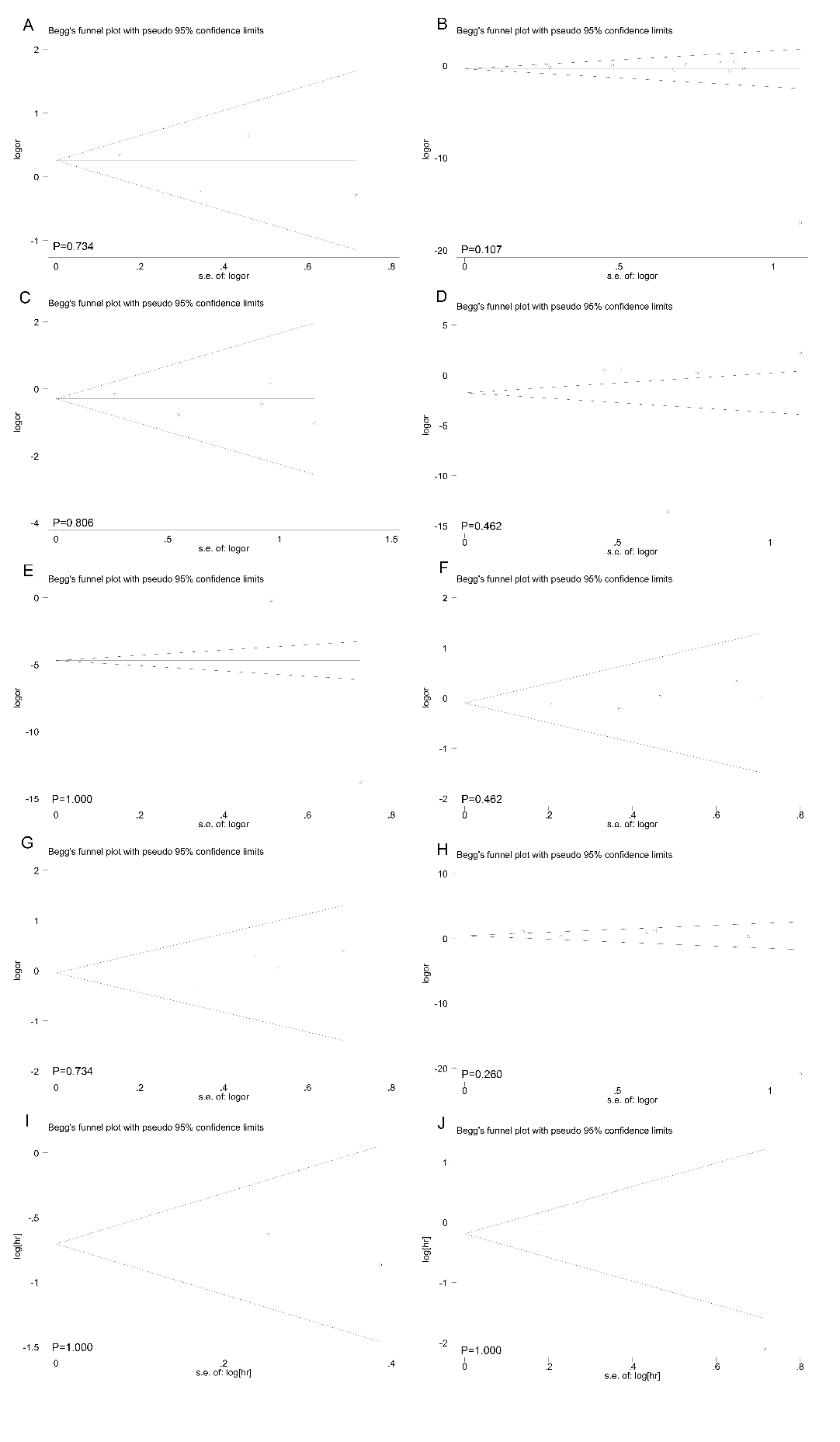
Supplementary Figure 1. Begg’s funnel plots of publication bias for the association between AR expression and prognostic factors. (A) Age; (B) Grade; (C) Lymph node status; (D) Myometrial invasion; (E) Cervical invasion; (F) Stage (I+II vs. III+Ⅳ); (G) Lymphovascular invasion; (H) Histological type (I vs. II); (I) Univariate survival analysis; (J) Multivariate survival analysis.
